# Our Contemporaries

**Published:** 1913-06

**Authors:** 


### OUR CONTEMPORARIES

---

The *Medical Times* for April has a valuable symposium on the medical curriculum.

---

The *Medical Review of Reviews* for March contains the following as part of one of its editorials:

"What is ethics for one should be ethics for all. While practitioners of high professional standing accept rebates, while they publish papers that have no original merit, while they continue to talk in medical meetings upon subjects about which they are not qualified to speak, while they give their views freely to the public, the force of medical ethics can hardly be said to be felt by them. A code of ethics is essential and desirable, but should above all be just and applicable to all members of the profession."

This reminds us that we were criticised, some time ago, for publishing a paper because the author was not one of the elect, although it was conceded that he was in good standing and had written a good article. A large share of the ethical problems and of the quarrels of the profession are due to the fact that men are not willing to accept a broad-minded, democratic, fraternal basis, but insist on a tacit recognition of a professional aristocracy and peasantry. And, the funny side of the matter is, that the trouble is made largely by those who, in ordinary society, would be considered members of the get-rich-quick aristocracy.

---

"Dying Words." One of our religious contemporaries prints a list of dying words of celebrated sinners, indicating a fear of Hell that should please the most orthodox. On the other hand, some time ago, we came across a quaint old book recording the dying words of some very pious Quakers, men, women and children, who died about a century ago. We want particularly to avoid the discussion of matters of dogma, but it is not without medical interest to know how frequently dying persons do utter the elaborate religious and other sentiments popularly supposed to be appropriate and customary, at the final leave taking. In our own experience—which we may say has been considerable, without either intending to boast of a large practice or to admit that the fatal termination was a logical result—dying persons have either been quite unconscious or, at least, too weak to express themselves except briefly and, for the most part, on commonplace matters. We do not remember ever to have witnessed the ecstacy of the good or the contrition and fear of the wicked which speakers on religious subjects so frequently mention. Perhaps our readers will think it worth while to state their experience.

